# Interaction of recommended levels of physical activity and protein intake is associated with greater physical function and lower fat mass in older women: Kuopio Osteoporosis Risk Factor- (OSTPRE) and Fracture-Prevention Study

**DOI:** 10.1017/S0007114520000045

**Published:** 2020-04-14

**Authors:** Samu Sjöblom, Joonas Sirola, Toni Rikkonen, Arja T. Erkkilä, Heikki Kröger, Sarang L. Qazi, Masoud Isanejad

**Affiliations:** 1 Kuopio Musculoskeletal Research Unit, University of Eastern Finland, Kuopio, Finland; 2 Department of Orthopedics and Traumatology, Kuopio University Hospital, Kuopio, Finland; 3 Institute of Public Health and Clinical Nutrition, University of Eastern Finland, Kuopio, Finland; 4 Department of Musculoskeletal Biology, Institute of Ageing and Chronic Disease, University of Liverpool, Liverpool, UK

**Keywords:** Nutrition, Protein intake, Physical activity, Skeletal muscle, Sarcopenia

## Abstract

The aim of the study was to investigate whether the interaction of physical activity (PA) and protein intake is associated with physical function (PF). The women from the Osteoporosis Risk Factor and Fracture Prevention Study (*n* 610) completed a questionnaire on lifestyle factors and PA and underwent PF and body composition measurements at baseline (BL) and over 3 years of follow-up (3y-FU). PA was categorised according to WHO cut-off PA = 0, 0 < PA < 2·5 and PA ≥ 2·5 h/week. Protein intake was calculated from the 3-d food record at baseline and categorised according to the Nordic Nutrition Recommendations <1·1 and ≥1·1 g/kg body weight (BW). The results showed in univariate ANOVA at the baseline and at the 3-year follow-up, women with high PA ≥ 2·5 h/week and protein intake ≥ 1·1 g/kg BW had higher grip strength adjusted for BMI, higher mean number of chair rises, faster mean walking speed, higher modified mean short physical performance battery score and lower mean fat mass compared with other interaction groups. High PA and protein intake were associated with lower BMI despite significantly higher energy intake. In conclusion, higher PA and protein intake interaction was associated with greater PF and lower fat mass, but the association with relative skeletal muscle index and muscle mass was inconclusive. The present study gives noteworthy information for preventing sarcopenia.

Ageing is accompanied with loss of muscle mass and muscle strength, which results diminished physical function (PF) and debilitating consequences such as fall and mortality in older people^(1)^.

The exact mechanism of PF decline in older people is yet to be investigated. In this context, identifying the most effective intervention strategy to preserve PF in older people has been of high public health interest^(2,3)^.

Among the most important health behaviours are those that involve physical activity (PA) and diet to preserve muscle mass and PF in older life^(4–6)^. Physical inactivity is linked to the loss of PF and muscle mass in older people^(7)^. Studies have shown that alterations in PF and muscle fibres in older adults remain responsive to functional demands such as physical exercise^(8)^. Therefore, it has been suggested that regular PA can partially prevent progression of the loss of PF and muscle mass related to inactivity^(9,10)^. The influence of PA on muscle has been described in relation to several of the factors acting on muscle in age-related imbalance processes, and exercise may up-regulate the metabolism of muscle synthesis^(8)^.

In addition, a growing body of evidence indicates that increased dietary protein intake in the older population may prevent the loss of PF and muscle mass^(11–13)^. Furthermore, increased dietary protein intake can enhance the effects of PA on functional ability^(14,15)^. In a study among adults, functional tasks that benefitted most from a higher-protein diet (≥1·2 *v*. <0·8 g/kg per d) were doing heavy work at home (e.g. shovelling, or washing windows, walls or floors), walking half a mile, going up and down stairs, stooping/kneeling/crouching and lifting heavy items^(16)^. The exact mechanism is unclear, but it has been suggested that exercise-induced improvement of protein synthesis may be due to nutrient-stimulated vasodilation and nutrient delivery to muscle^(17)^. Although the interaction effect of PA and protein intake may prevent the decline of muscle mass and PF in older people, observational studies are scarce^(5)^.

Most previous studies have evaluated the association of PA and protein intake using population cut-offs (*n*-tiles) or as a continuous variable. Although this approach is warranted, formulating PA and protein intake recommendations for older people, to preserve PF and muscle mass based on the results, is somewhat challenging. The WHO has recommended that adults aged 65 years and older ‘should do at least 150 min of moderate-intensity aerobic PA throughout the week to improve cardiorespiratory and muscular fitness, bone and functional health, reduce the risk of non-communicable disease, depression and cognitive decline’^(18)^. Furthermore, the Nordic Nutrition Recommendations (NNR 2012) outline a protein intake in the range of 1·1–1·3 g/kg BW (minimum 1·1 g/kg BW) to preserve PF in older adults^(19)^. However, data from observational studies using such recommendations for PA and protein intake in older adults are scarce.

In the present study, we investigated the association of PA independently and in an interaction with protein intake in relation to PF and muscle mass in older postmenopausal women. This approach provided an opportunity to study PA according to the WHO cut-off and protein intake according to the NNR 2012 recommendations.

## Materials and methods

### Study population

Data for the present study were collected from the Osteoporosis Risk Factor and Prevention – Fracture Prevention Study (OSTPRE-FPS), which began in 2003 in Kuopio, Finland^(20)^. OSTPRE-FPS was a randomised population-based double-blinded controlled trial with a 3-year follow-up involving 3432 women (aged 66–71 years). The primary aim of the study was to determine whether vitamin D and Ca supplementation would be effective in preventing falls and fractures in postmenopausal women.

The power analysis was performed based on the incidence of fractures^(20)^. A subsample of 750 women was randomly selected at the baseline (the supplementation and control groups both had 375 subjects) and underwent body composition, clinical, physical and laboratory tests. There was no *a priori* power analysis to calculate the size of the subsample of 750 women randomly selected from the 3432 women at the baseline.

The baseline measurements took place between February 2003 and May 2004 and the follow-up measurements between January 2006 and May 2007 (follow-up time 2·8 (sd 0·4) years)^(21)^. Between the randomisation and the actual start of the intervention, 237 subjects withdrew and ten women stopped participating in the study for various reasons during the 3-year follow-up period. At the end of the trial (*n* 593), 306 and 287 subjects in the intervention and control groups of the subsample, respectively, completed the follow-up. The present study was a *post hoc* analysis of the subjects from the OSTPRE-FPS study. The final analytical data set for the present study comprised 608 women who had baseline and follow-up data for the self-administered questionnaire regarding PA. However, the 3-d dietary food record was only available at the baseline for 554 women (data for fifty-four food records were missing due to not returning the food record or returning an incomplete one, which resulted in missing values).

All clinical measurements were performed in the Kuopio Musculoskeletal Research Unit of the Clinical Research Centre of the University of Kuopio. All participants provided written permission for participation. The study was approved in October 2001 by the ethical committee of Kuopio University Hospital. The study was registered in Clinical trials.gov by the identification NCT00592917.

### Questionnaires

The OSTPRE-FPS baseline questionnaire in 2003–2004 contained questions on income per month (euros), age at menopause (years), chronic diseases and years of hormone therapy. In addition, questions about current smoking and tobacco use (no/yes), previous falls, prescribed medications and use of self-reported vitamin D and Ca supplements were asked.

### Body composition measurements

Total body dual-energy X-ray absorptiometry was used by specially trained nurses to measure muscle mass and fat mass (FM) in 2003–2007. The dual-energy X-ray absorptiometry measurements were carried out using the same Lunar Prodigy, adhering to the imaging and analysis protocols provided by the manufacturer (Lunar Co.)^(22)^. The height and weight of participants were measured in light indoor clothing without shoes at the baseline and at the 3-year follow-up, and BMI was calculated as kg/m^2^. Appendicular lean mass is the sum of lean mass in arms and legs, whereas the relative skeletal muscle index (RSMI) was calculated as appendicular lean mass divided by height squared (kg/m^2^).

### Physical function measurements

The detailed explanation of PF measurements in these data has been published previously^(23,24)^. PF measures were assessed by trained nurses at the baseline and at the 3-year follow-up session, including hand grip strength (kg), number of chair rises in 30 s, ability to squat, knee extension (Newtons but converted into kg), walking speed 10 m (m/s), tandem walk for 6 m (m/s), standing with closed eyes for 10 s and one leg stance performance for 30 s. The follow-up variable of knee extension was excluded from the analysis because of an unexpected increase in measured extension force and/or possible data entry errors, which could not be traced due to the long period between this secondary analysis and the time data recorded in 2001–2003. This issue was explained in these data^(24)^. Grip strength (kg) was measured using the dominant hand while sitting on a bench, with the forearm flexed from the elbow at a 90-degree angle, near the torso. A total of three attempts were recorded, with approximately 30 s of resting time between the tests. Close attention was paid to making all three attempts in a similar, fixed posture (JAMARTM handgrip dynamometer; Sammons Preston), and then mean grip strength was calculated. The interaction correlation coefficient for grip strength measurements was 0·93. To standardise the measurement, grip strength was further expressed as a ratio to the BMI. The chair rise test was conducted if the participant could rise at least once without using their arms from a straight-backed, non-padded, armless chair. The maximum number of chair rises was recorded by trained nurses. The women were asked to walk the 10-m distance first at normal pace and then the 6-m distance in a tandem position. The time was recorded, and the walking speed was calculated as m/s. The women who were not able to walk were given a value of zero (fourteen women at the baseline and twenty-five women in the 3-year measurement).

The short physical performance battery (SPPB) may indicate an individual’s physical ability^(25)^. The modified SPPB score was calculated based on the European Working Group on Sarcopenia definition. A modified SPPB was calculated using three individual measures of PF assessment, including: (1) walking speed over 10 m (m/s), (2) chair rises in 30 s and (3) standing on one leg. These three PF variables were further categorised into quartiles, and each quartile was scored on a scale of 1–4 points. Individuals unable to complete a task received a score of 0. The total SPPB score ranged from 0 to 12, with higher scores referring to better physical performance.

### Physical activity

Within the baseline questionnaires, PA data were collected on the amounts of different types of household, leisure activities and exercise engaged in, including winter and summer seasons at the baseline and over 3 years. Household and leisure activities that were asked included wood work, hunting, fishing, major work at home such as renovation and house chores. The exercise that women reported included walking, cycling, skiing, swimming, aerobic exercise, ball sports (e.g. football, volleyball, indoor hockey, etc.), skating, bowling, floorball, gymnastics and rowing. However, among the household, leisure and exercise variables, the most common activities reported were skiing, walking, cycling, swimming and aerobic exercise, explaining over 90 % of the weekly PA in these data (data not shown), which was used to form the PA variable. These types of activities are normally referred to as aerobic exercise. However, because the intention of our questionnaire at the time of data collection was to estimate on overall PA level, we refer to these activities as PA rather than aerobic exercise. The reported amount of weekly PA at those time points was used to form a long-term PA variable by summing up the average weekly PA at the baseline.

Our secondary analysis showed that there was a significant deviation in reported intensity within- and between-study subjects. Therefore, the intensity of exercise was introduced to models as a covariate rather than forming the final PA variable. The women were then categorised into the following groups: inactive (0 h/week), insufficient (0 < PA < 2·5 h/week) and sufficient (PA ≥ 2·5 h/week); the cut-offs were adapted according to WHO recommendations for amount of PA per week in older adults^(18)^. In addition, the continuous PA variable was introduced in one-unit increments to account for normality. In NNR 2012, the recommendation for PA is identical to the WHO recommendation, which is 150 min of moderate-intensity PA per week. Examples that have been stated by the WHO and NNR include climbing stairs, walking 4·8 km/h, snow clearing, lawn mowing, dancing, gardening, hiking and swimming.

### Dietary and protein intake

Dietary intake was assessed using a 3-d food record at the baseline. A questionnaire and instructions were sent to participants beforehand, and they were returned at the baseline visit. Participants were instructed to record their diet and everything they ate and drank, and to evaluate the amount of food using household measures for 3 consecutive days, with 2 d during the week and 1 d during the weekend (Saturday or Sunday)^(26)^. In the case of uncertainties in the food record, a nutritionist called the participant for more information. Nutrient intakes were calculated using Nutrica dietary analysis software (version 2.5, Finnish Social Insurance Institute), based on the national database of the Finnish Social Insurance Institution. Assessment of underreporting has previously been described, and no participants were excluded due to low energy intake^(24)^. Total protein intake was calculated first as g/d and was then further expressed as g/kg BW. In these data, we previously introduced the cut-offs for protein intake according to NNR 2012^(19)^. In the present study, accordingly, women were categorised by protein intake (g/kg BW) according to NNR 2012 (<1·1 *v*. ≥1·1 g/kg BW).

### Confounders

Data regarding lifestyle were self-reported at the baseline. The variables of interest included income per month (euros), marital status (married, divorced, widowed and not married), smoking status (never, past and current), medical history (diseases and surgeries), medications (including hormone therapy) and time since menopause^(21)^. Number of chronic diseases, including hypertension, hyperlipidaemia, CHD, diabetes, arthritis, osteoporosis, depression, chronic kidney disease and cancer were reported. Self-ambulatory status was defined as normal if women were (a) fully capable of moving, (b) capable of moving but unable to run or (c) capable of walking 1 km at the most. The status was defined as restricted when women were (a) capable of walking 100 m at the most, (b) moving only indoors or (c) incapable of moving.

### Statistical analysis

All statistical analyses were executed using SPSS software version 25 for Windows (IBM Corp.). All tests were two-sided, and a *P* value of <0·05 was considered significant. To account for the possible intervention effect, we conducted preliminary analyses and there was neither a significant effect on the outcome of interests nor an interaction with PA and protein intake. Thus, data were pooled for the total population (intervention and control group) in year 3 of the follow-up. However, follow-up analyses were further adjusted for the randomised controlled trials (RCT) study group (Ca and vitamin D intervention). Pearson’s correlation coefficient *r* was calculated for the relationships between PA, protein intake, the interaction between these variables and PF measures. The PF measures *r*^2^ accounted for by the three independent variables (i.e. PA, protein intake and PA interaction with protein intake) were calculated using multiple regression analysis (presented in online Supplementary Table S1). This computation provided an estimate of the respective contribution of the three independent variables to the PF measures.

Baseline characteristics were tested using ANOVA for continuous variables and *χ*^2^ test for categorical variables, according to PA groups (0, 0 < PA < 2·5, and PA ≥ 2·5 h/week) stratified for protein intake cut-off (<1·1 and ≥1·1 g/kg BW). For the main analysis, PA was introduced to two main models, first as a categorical variable to univariate ANOVA (UNIANOVA) for calculated means and standard deviations, and second as a continuous variable in multiple regression analysis for calculated standard *β-*coefficient and standard error. SPSS software provided the multiple regression, which is an extension of simple linear regression. This model could predict the value of the outcome variable (PF and muscle mass) based on the values of multiple independent variables, including independent variables (PA alone and in interaction with protein intake) and covariates^(27)^. The outcome variables were PF, total body lean mass, RSMI and total body FM at baseline, at 3 years, and for their absolute changes over 3 years of follow-up.

### Interaction analysis of physical activity and protein intake

An interaction term was introduced by multiplying the continuous variables of PA h/week and protein intake g/kg BW, and this was used as an independent variable in multiple regression analyses, with multiple PF measures and muscle mass as dependent variables. The interaction was statistically significant (*P* < 0·040). For interaction analysis, we merged the two groups of PA = 0 and 0 < PA < 2·5 h/week into one, as PA < 2·5, to provide a balanced number of subjects in each group, because the initial descriptive analysis showed only four women belonging to the PA = 0 and protein ≥1·1 g/ kg BW group. The final four interaction groups were: (a) PA < 2·5 and protein intake <1·1 g/kg BW, (b) PA < 2·5 and protein intake ≥1·1 g/kg BW, (c) PA ≥ 2·5 h/week and protein intake <1·1 g/kg BW and (d) PA ≥ 2·5 h/week and protein intake ≥1·1 g/kg BW. Interaction between continuous PA at 1-h intervals and protein intake was significant in relation to FM, and PF measures (*β* > 0·75, *P* < 0·040), except for the ability to squat to the ground and tandem walk speed.

UNIANOVA was used to calculate mean values and standard deviations (PA and protein intake interaction as a categorical variable), and multiple regression analysis was used to calculate the standard *β-*coefficient and SE (PA and protein intake interaction as a continuous variable) in the models, where PF measures, total body LM, total body FM and RSMI were set as dependent variables.

The final analytical sample for interaction analysis was performed for 554 women because there were fifty-four (total number = 608) women without a dietary food record and they could not be included in the interaction analysis. However, our prior analysis showed no significant differences in the baseline characteristics variables (presented in Table 1) between those that were and were not included in the interaction analyses (data not shown).

**Table 1.** Baseline characteristics and dietary factors of the participants according to physical activity (PA) (h/week) groups* (Mean values and standard deviations; numbers and percentages)

	Inactive: PA = 0 h/week (*n* 77)			Insufficient: 0 < PA < 2·5 h/week (*n* 166)			Sufficient: PA ≥ 2·5 h/week (*n* 365)			
	Mean		sd	Mean		sd	Mean		sd	*P*
PA (h/week)	0		0	1·2		0·69	6·1		4·7	0·0001
PA range (h/week)		0–0			1·1–1·3			5–6		0·0001
Age (years)	67·7		1·9	68·0		1·8	67·7		1·8	0·415
BMI (kg/m^2^)	29·0		4·7	27·9		4·5	26·8		3·8	0·0001
Time since menopause (years)	19·4		7·4	18·6		5·2	18·4		5·4	0·364
Income (euros/month)	787		269	845		308	880		294	0·173
Number of chronic diseases	1·4		1·0	1·5		1·2	1·4		1·1	0·774
Mobility status										0·211
Normal										
*n*		40			136			324		
%		80·0			88·9			96·1		
Restricted										
*n*		10			17			13		
%		20·0			11·1			3·9		
Current smoker										0·596
*n*		2			10			14		
%		3·8			6·5			4·2		
Current hormone therapy										0·411
*n*		8			35			80		
%		15·1			22·6			23·3		
Dietary factors and food groups										
Energy intake (kJ)	5816		1494	6552		1498	6665		1556	0·001
Alcohol (g/d)	6·0		4·2	9·0		4·6	10·9		3·7	0·111
Protein (g/d)	59·7		17·9	68·1		16·8	69·2		18·3	0·001
Protein (g/kg body weight)	0·78		0·25	0·95		0·25	0·99		0·27	<0·0001
Carbohydrate (g/d)	174·6		47·6	192·7		45·9	196·1		49·0	0·007
Fat (g/d)	47·2		16·4	56·4		18·7	17·9		0·96	0·019

* ANOVA was used to calculate means and standard deviations, and *χ*^2^ tests for categorical variables to calculate *n* and percentages according to PA groups. *P* values are significant two-tailed.

### Covariates and adjustment for analytical models

We initially assessed known covariates of sarcopenia based on the literature. Furthermore, covariates were selected based on their multicollinearity and their predictive values alone, which led to the selection of the following models. For both multiple regression analysis and UNIANOVA, the first model was adjusted for age and energy intake (kJ). The second model was adjusted for the variables in model 1 plus smoking (yes, no), hormone therapy (yes, no), rheumatoid arthritis, baseline height (m), income per month (euros) and intensity of PA. Follow-up analyses were adjusted for changes in PF variables and RCT study group (intervention with Ca and vitamin D supplementation).

## Results

Table 1 shows the baseline characteristics according to PA groups. PA was reported to be equal or higher than 2·5 h/week in 365 women. Those with PA ≥ 2·5 h/week had significantly lower BMI and FM. Energy and protein intake were both higher in women with higher PA. The baseline characteristics according to the PA and protein intake interaction groups are reported in Table 2. High PA and protein intake were associated with lower BMI and higher energy intake. Half of the women received Ca (1000 mg) and vitamin D (17·5 μg) during the 3-year follow-up. Results showed no significant effect of vitamin D and Ca supplementation on PF or muscle mass between the intervention and control group.

**Table 2.** Baseline characteristics and dietary factors of the participants according to physical activity (PA) (h/week) groups stratified for protein intake cut-off* (Mean values and standard deviations; numbers and percentages)

	Protein intake < 1·1 g/kg body weight									Protein intake ≥ 1·1 g/kg body weight									
	PA = 0 h/week (*n* 45)			0 < PA < 2·5 h/week (*n* 111)			PA ≥ 2·5 h/week (*n* 231)			PA = 0 h/week (*n* 4)			0 < PA < 2·5 h/week (*n* 50)			PA ≥ 2·5 h/week (*n* 113)			
	Mean		sd	Mean		sd	Mean		sd	Mean		sd	Mean		sd	Mean		sd	*P*
Age (years)	67·9		2·2	67·9		1·8	67·8		1·8	67·9		2·0	67·9		1·8	67·8		1·8	0·553
BMI (kg/m^2^)	30·5		4·5	29·1		4·6	28·6		4·0	30·4		4·5	29·1		4·6	27·6		4·0	0·001
Time since menopause (years)	19·4		7·6	18·4		4·9	18·4		5·2	19·2		7·1	19·1		6·2	17·5		4·7	0·282
Income (euros/month)	747		286	831		309	879		297	987		255	880		339	893		292	0·734
Number of chronic diseases	1·5		1·0	1·5		1·1	1·5		1·2	1·7		0·8	1·4		1·2	1·3		1·0	0·409
Mobility status																			
Normal																			0·0001
*n*		32			93			212			8			43			112		
%		76·2			84·5			94·6			100			100			99·1		
Restricted																			
*n*		10			17			12			0			0			1		
%		23·8			15·5			5·4			0			0			0·9		
Current smoker	2		4·7	9		8·2	13		5·8	0		0	1		2·3	1		0·9	0·644
*n*																			
%																			
Current hormone therapy																			0·472
*n*		7			26			55			1			9			25		
%		15·6			23·4			23·8			12·5			20·5			22·1		
Dietary factors and food groups																			
Energy intake (kJ)	5581		1473	6029		1230	6113		1343	7150		741	7870		1289	7803		1335	0·025
Alcohol (g/d)	4·8		1·2	7·7		1·1	10·7		1·8	12·3		1·5	12·3		1·5	11·3		17	0·044
Protein (g/d)	55·7		15·6	61·8		11·9	60·5		13·4	81·8		13·7	86·2		14·5	86·9		13·6	0·033
Protein (g/kg body weight)	0·69		0·2	0·81		0·14	0·83		0·16	1·2		0·1	1·3		0·2	1·3		0·2	0·045
Carbohydrate (g/d)	168		47	179		40	182		42	214		33·3	224		42	226		48	0·137
Fat intake (g/d)	46·2		15·5	49·6		15·8	50·3		16·9	55·6		16·9	63·9		17·2	226		48	0·171

* ANOVA was used to calculate means and standard deviations, and *χ*^2^ tests for categorical variables to calculate *n* and percentages according to physical activity groups stratified for protein intake.

Univariate analysis indicated that, for PF and muscle mass measures, the most significant correlation was with PA and the protein intake interaction value (online Supplementary Table S1). PA was not associated with lean mass and RSMI, whereas protein intake was inversely associated with lean mass and RSMI.

### Physical activity and physical function

In cross-sectional analysis, continuous PA at 1-h intervals was positively associated with higher number of chair rises (standardised *β* 0·248, *P* = 0·040), walking speed (standardised *β* 0·107, *P* = 0·005), modified SPPB (standardised *β* 0·118, *P* = 0·022), ability to squat (standardised *β* 0·185, *P* = 0·001) and lower FM (standardised *β* −0·196, *P* < 0·001). Similarly, the cross-sectional analysis results of UNIANOVA showed that the means for walking speed, standing on one leg and modified SPPB were significantly higher in the high PA group as a categorical variable compared with the low PA group after adjusting for confounders. At the 3-year follow-up, higher continuous PA (1-h intervals and PA ≥ 2·5 h/week) was significantly associated with walking speed, ability to squat and lower FM (*P* < 0·048) (Table 3).

**Table 3.** Physical function and body composition in physical activity (PA) (h/week) groups* (Mean values and standard deviations; *β* values with their standard errors)

	PA = 0 h/week (*n* 77)		0 < PA < 2·5 h/week (*n* 166)		PA ≥ 2·5 h/week (*n* 365)		PA at 1-h intervals (*n* 608)				
	Mean	sd	Mean	sd	Mean	sd	*β*	s e	*P* †	*P* ‡	*P* §
Hand grip strength (kg)											
Baseline	26·0	5·1	25·6	4·7	25·8	4·8	–0·006	0·060	0·391	0·501	0·886
At 3 years||	25·2	6·3	24·8	6·1	24·7	5·5	–0·031	0·058	0·698	0·478	0·443
Hand grip strength adjusted for BMI (kg/m^2^)											
Baseline	0·90	0·24	0·93	0·22	0·97	0·21	–0·007	0·060	0·387	0·486	0·867
At 3 years||	0·90	0·24	0·93	0·22	0·97	0·21	–0·044	0·002	0·211	0·651	0·787
Knee extension (Newtons)											
Baseline	275	89	288	82	302	82	0·079	0·986	0·118	0·121	0·075
Chair rises in 20 s											
Baseline	6·2	3·0	7·5	2·6	8·0	2·6	0·248	0·047	0·047	0·046	0·040
At 3 years||	7·2	4·2	8·1	3·1	9·2	3·1	0·002	0·031	0·078	0·077	0·098
Tandem walk over 6 m (s)											
Baseline	17·0	10·0	19·4	8·1	19·6	8·5	0·027)	0·003	0·605	0·349	0·549
At 3 years||	19·8	4·4	20·4	7·0	20·1	10·4	0·038	0·153	0973	0·588	0·406
Walking speed over 10 m (m/s)											
Baseline	1·4	0·2	1·5	0·2	1·7	0·2	0·107	0·004	0·015	0·012	0·005
At 3 years||	1·4	0·2	1·5	0·3	1·6	0·3	0·017	0·311	0·099	0·050	0·048
Standing on one leg for maximum 30 s (s)											
Baseline	13·2	11·1	17·6	10·2	20·2	9·1	0·113	0·105	0·011	0·124	0·095
At 3 years||	15·2	12·0	17·0	11·5	17·8	10·6	0·057	0·097	0·114	0·250	0·194
Modified short physical performance battery (calculated score)											
Baseline	5·2	1·5	5·8	1·7	6·4	1·9	0·118	0·046	0·005	0·065	0·022
At 3 years||	7·8	2·4	7·6	1·6	7·6	1·7	0·009	0·021	0·387	0·433	0·840
Able to perform squat (% of women)											
Baseline	43		71		72		0·185	0·002	0·027	0·039	0·001
At 3 years||	76		89		93		0·039	0·004	0·001	0·005	0·007
Lean mass (kg)											
Baseline	41·2	4·0	40·4	4·3	39·9	4·2	0·045	0·037	0·654	0·733	0·191
At 3 years||	41·3	4·9	40·7	4·7	40·3	4,6	0·046	0·039	0·718	0·882	0·154
Relative skeletal muscle index (kg/m^2^)											
Baseline	6·8	0·7	6·8	0·6	6·7	0·6	0·047	0·017	0·258	0·214	0·180
At 3 years||	6·6	0·9	6·7	0·6	6·6	0·6	0·097	0·008	0·419	0·517	0·318
Fat mass (kg)											
Baseline	33·1	10·0	30·0	7·	27·4	8·4	–0·196	0·222	0·001	0·001	0·0001
At 3 years||	31·8	12·1	30·1	8·4	27·6	8·9	–0·140	0·237	0·018	0·020	0·014

UNIANOVA, univariate ANOVA.

* UNIANOVA was used to calculate mean values and adjusted standard deviations. Multiple regression analysis was used to calculate *β*-coefficient and standard error. Model 1 was adjusted for age and energy intake. Model 2 was adjusted for age, energy intake (kJ), smoking (yes, no), hormone therapy (yes, no), rheumatoid arthritis, baseline height (m), income per month (euros) and intensity of PA.

† Mean values and standard deviations calculated from UNIANOVA adjusted for variables in model 1.

‡ Mean values and standard deviations calculated from UNIANOVA adjusted for variables in model 2.

§ *P* calculated from multiple regression analysis, regression coefficient adjusted for variables in model 2.

|| At 3 years of follow-up, analyses were adjusted for absolute changes in physical function, muscle mass and study group (Ca and vitamin D intervention).

### Physical activity and protein intake interaction

At the baseline, after adjusting for the confounders in regression analysis, continuous PA at 1-h intervals and protein intake interaction were associated with higher grip strength adjusted for BMI (*β* 0·140 and *P* = 0·001), faster walking speed over 10 m (*β* 0·205 and *P* < 0·001), longer standing on one leg for a maximum of 30 s (*β* 0·106 and *P* = 0·014), more frequent ability to squat (*β* 0·118 and *P* = 0·050), higher modified SPPB score (*β* 0·177, se = 0·003 and *P* < 0·001) and lower FM (*β* −0·206, and *P* < 0·001) (Table 4). Follow-up analysis further showed that continuous PA at 1-h intervals and protein intake interaction were associated with higher grip strength adjusted for BMI (*β* 0·087, se = 0·003 and *P* = 0·046), increased repetitions of higher number of chair rises (*β* 0·190, se = 0·004 and *P* = 0·001), faster walking speed over 10 m (*β* 0·143 and *P* = 0·002), more frequent ability to squat (*β* 0·105 and *P* = 0·022), higher modified SPPB score (*β* 0·089 and *P* = 0·043) and lower FM (*β* −0·198 and *P* < 0·001). The only significant association of PA and protein intake interaction as a continuous variable was with FM change (*β* −0·095 and *P* = 0·040).

**Table 4.** Association of physical activity (PA) and protein intake interaction as categorical and continuous variables with physical function assessment at baseline and 3-year follow-up* (Mean values and standard deviations; *β* values with their standard errors)

	PA < 2·5 h/week; protein < 1·1 g/kg g/kg BW (*n* 147)			PA < 2·5 h/week; protein ≥ 1·1 g/kg g/kg BW (*n* 46)			PA ≥ 2·5 h/week; protein < 1·1 g/kg BW (*n* 240)			PA ≥ 2·5 h/week; protein ≥ 1·1 g/kg BW (*n* 119)			PA and protein intake interaction continuous variable (*n* 552)				
	Mean		sd	Mean		sd	Mean		sd	Mean		sd	*β*	se	*P* †	*P* ‡	*P* §
Hand grip strength (kg)																	
Baseline	26·1		0·5	24·2		0·9	25·8		0·4	24·1		0·6	0·020	0·125	0·282	0·205	0·638
At 3 years	24·9		6·1	25·0		5·6	25·2		4·8	24·1		5·0	–0·008	0·05	0·718	0·896	0·852
Change	–1·0		0·42	0·34		0·78	–1·15		0·32	–0·29		0·47	–0·030	0·053	0·274	0·250	0·511
Hand grip strength adjusted for BMI (kg/m^2^)																	
Baseline	0·89		0·02	1·0		0·4	0·95		0·1	1·1		0·2	0·140	0·003	0·019	0·047	0·001
At 3 years	0·83		0·25	0·88		0·24	1·00		0·25	0·92		0·20	0·087	0·003	0·001	0·001	0·046
Change	–0·04		0·01	0·02		0·03	–0·05		0·01	0·04		0·01	–0·069	0·002	0·083	0·046	0·139
Knee extension (Newtons)																	
Baseline	291		7·8	277		13·2	304		5·9	289		8·5	0·090	0·929	0·211	0·115	0·054
Chair rises in 20 s																	
Baseline	6·9		0·3	8·2		0·5	7·8		0·2	8·6		0·3	0·050	0·048	0·035	0·048	0·273
At 3 years	7·7		3·1	8·6		3·1	8·4		3·3	9·8		2·6	0·190	0·030	0·008	0·002	0·001
Change	0·01		0·04	0·00		0·07	0·02		0·03	–0·09		0·03	0·088	0·045	0·913	0·897	0·057
Tandem walk speed over 6 m (m/s)																	
Baseline	0·29		0·04	0·34		0·07	0·35		0·03	0·32		0·04	0·049	0·003	0·603	0·779	0·304
At 3 years	0·31		0·12	0·33		0·11	0·33		0·10	0·33		0·10	0·048	0·142	0·441	0·489	0·302
Change	–0·07		0·04	–0·02		0·07	–0·03		0·03	0·03		0·04	–0·042	0·004	0·786	0·788	0·396
Walking speed over 10 m (m/s)																	
Baseline	1·50		0·03	1·73		0·06	1·69		0·02	1·79		0·04	0·205	0·004	<0·001	0·010	<0·001
At 3 years	1·47		0·03	1·54		0·02	1·55		0·05	1·62		0·03	0·143	0·004	0·027	0·039	0·002
Change	–0·08		0·03	–0·17		0·05	–0·11		0·02	–0·13		0·03	–0·586	0·004	0·570	0·486	0·558
Standing on one leg for maximum 30 s																	
Baseline	15·6		1·0	19·2		2·0	19·0		0·8	22·0		1·2	0·106	0·110	0·002	0·012	0·014
At 3 years	16·1		0·9	16·8		0·7	18·0		1·5	20·1		1·1	0·098	0·017	0·071	0·078	0·289
Change	–0·3		1·2	–0·2		2·2	–2·3		0·9	–3·0		1·4	–0·012	0·118	0·418	0·361	0·801
Able to perform squat (% of women)																	
Baseline		55			77			67			93		0·118	0·002	0·003	0·048	0·050
At 3 years		83			88			91			94		0·105	0·003	0·241	0·113	0·022
Change		20			–15			29			–10		0·057	0·004	0·343	0·330	0·218
Modified SPPB (calculated score)																	
Baseline	5·4		1·7	6·2		1·9	6·2		1·5	6·5		1·7	0·177	0·040	<0·001	<0·001	<0·001
At 3 years	7·3		1·8	7·4		1·8	7·6		1·6	8·2		1·6	0·089	0·020	0·018	0·010	0·043
Change	1·8		2·2	1·2		2·2	1·4		1·8	1·5		2·1	–0·125	0·027	0·112	0·074	0·098
Lean mass (kg)																	
Baseline	41·0		4·5	38·8		3·5	40·4		4·3	39·0		3·8	0·096	0·022	0·685	0·710	0·121
At 3 years	41·2		5·0	40·3		4·5	38·9		2·5	39·1		4·0	0·178	0·012	<0·001	<0·001	0·311
Change	0·2		0·1	–0·1		0·2	0·07		0·11	0·07		0·17	–0·025	1·82	0·432	0·421	0·592
RSMI (kg/m^2^)																	
Baseline	6·5		0·4	6·7		0·6	6·8		0·5	7·0		0·6	0·069	0·007	<0·001	<0·001	0·127
At 3 years	6·8		0·8	6·6		0·6	6·4		0·5	6·6		0·6	–0·057	0·008	<0·001	<0·001	0·277
Change	0·0		0·4	0·1		0·2	–0·1		0·3	0·0		0·3	0·023	0·005	0·434	0·537	0·626
Fat mass (kg)																	
Baseline	33·6		8·2	23·8		6·6	29·4		8·4	24·2		6·2	–0·206	0·089	<0·001	<0·001	<0·001
At 3 years	33·0		9·4	29·7		8·6	22·9		6·5	23·8		6·3	–0·198	0·097	<0·001	<0·001	<0·001
Change	0·05		3·12	0·08		3·21	–0·94		2·31	–0·19		2·18	–0·095	3·431	0·487	0·605	0·040

BW, body weight; SPPB, short physical performance battery; RSMI, relative skeletal muscle index; UNIANOVA, univariate ANOVA.

* UNIANOVA was used to calculate mean values and standard deviations adjusted. Multiple regression analysis was used to calculate *β-*coefficient and standard error. Model 1 was adjusted for age and energy intake. Model 2 was adjusted for age, energy intake (kJ), smoking (yes, no), hormone therapy (yes, no), rheumatoid arthritis, baseline height (m), income per month (euros) and intensity of PA. At 3 years of follow-up, analyses were adjusted for absolute changes in physical function, muscle mass and study group (Ca and vitamin D intervention).

† Mean values and standard deviations calculated from UNIANOVA adjusted for variables in model 1.

‡ Mean values and standard deviation calculated from UNIANOVA adjusted for variables in model 2.

§ Calculated from multiple regression analysis, regression coefficient adjusted for variables in model 2.

**Fig. 1.**
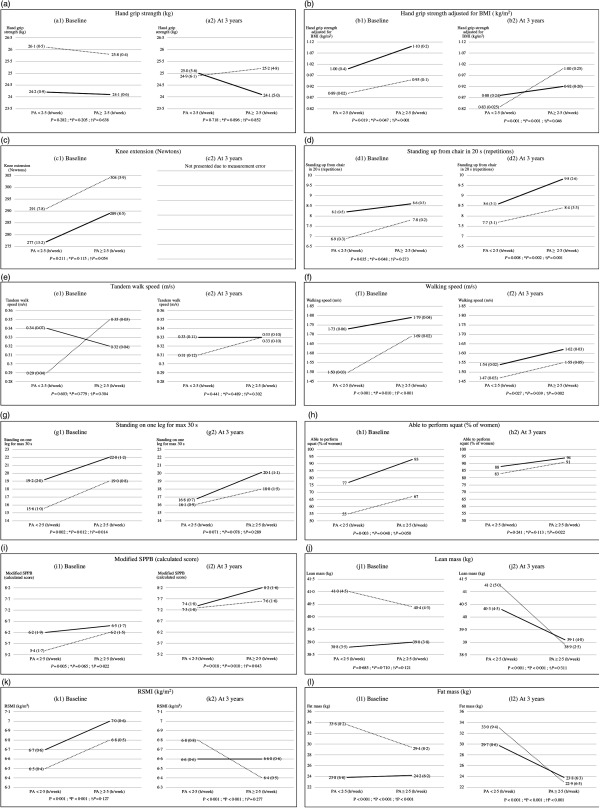
Univariate ANOVA (UNIANOVA) was used to calculate mean values and adjusted standard deviations. Multiple regression analysis was used to calculate *β*-coefficients and standard errors for physical function measures, lean mass, relative skeletal muscle index (RSMI) and fat mass according to physical activity (PA) and protein intake interaction groups: activity and protein intake interaction groups as follows: (i) PA < 2·5 h/week and protein intake <1·1 g/kg body weight (BW) (*n* 147); (ii) PA < 2·5 h/week and protein intake ≥1·1 g/kg BW (*n* 46); (iii) PA ≥ 2·5 h/week and protein intake <1·1 g/kg BW (*n* 240); (iv) PA ≥ 2·5 h/week and protein intake ≥1·1 g/kg BW (*n* 119). Model 1 was adjusted for age and energy intake. Model 2 was adjusted for age, energy intake (kJ), smoking (yes, no), hormone therapy (yes, no), rheumatoid arthritis, baseline height (m), income per month (euros) and intensity of PA. At 3 years of follow-up, analyses were adjusted for absolute changes in physical function, muscle mass and study group (calcium and vitamin D intervention). *P* calculated from UNIANOVA adjusted for variables in model 1. * *P* calculated from UNIANOVA adjusted for variables in model 2. † *P* calculated from multiple regression analysis adjusted for variables in model 2. The follow-up variable of knee extension was excluded from the analysis because of an unexpected increase in measured extension force and/or possible data entry errors, which could not be traced due to the long period between this secondary analysis and the time data recorded in 2001–2003. This issue was explained in these data^(24)^. 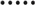, Protein < 1·1 (g/kg BW); 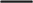, protein ≥ 1·1 (g/kg BW).

Further, in UNIANOVA at the baseline and at the 3-year follow-up, women with high PA ≥ 2·5 h/week and protein intake ≥1·1 g/kg BW had higher grip strength adjusted for BMI mean (1·1 (sd 0·2)), higher mean number of chair rises, faster mean walking speed, higher modified mean SPPB score and lower mean FM (Table 4, and Fig. 1) compared with the other interaction groups.

Women with highest PA and protein intake had significantly greater RSMI at the baseline (*P* < 0·001). To the contrary, at 3 years of follow-up, highest value for RSMI and LM was detected for women with lowest PA and protein intake (*P* < 0·001). When using continuous variables for PA and protein intake, the association with LM and RSMI was not significant for baseline or at 3 years of follow-up.

## Discussion

The present study provides an opportunity to assess the interaction of PA according to the WHO recommendation and protein intake according to NNR with muscle mass and PF. The foregoing analysis indicates that the interaction of PA 2·5 h/week with protein intake ≥1·1 g/kg BW was positively associated with PF at most measured sites and lower FM in older women. The results remained significant after adjusting for a number of relevant confounders. Significant associations in the prospective analyses remained at 3-years follow-up with walking speed, squat ability and lower FM, possibly due to small changes in the outcome measures over a relatively short follow-up (3 years). The interaction of PA and protein intake was not clearly associated with RSMI and lean mass. Previous epidemiological studies have showed that quadriceps, grip strength, ability to squat and walking speed have been determined as significant predictors of mortality^(28–30)^, and ability to squat and walking speed were associated with higher risk of fracture^(31)^.

There might be several mechanisms underlying the decline in PF in older people. Reduced strength with age may be a result of a combination of loss of muscle mass and neural control^(32)^. Ageing muscles are susceptible to reduced number of motor neurons which are mainly responsible of generating muscle strength^(33)^. In addition, muscle strength and PF in older people can be influenced by the hormonal changes such as insulin-like growth factor-I, which declines with age^(34,35)^.

### Physical activity and interaction of physical activity and protein intake with physical function

Adequate PA has been considered as an effective way to prevent PF decline in older people, but previous epidemiological studies defined PA different to our study as well as using different cut-offs for PA^(36–38)^, which should be noted. For instance, in a cohort study by Hillson *et al*., PA was self-reported, by frequency of participation in ‘mildly energetic’ (e.g. walking, weeding, general housework); ‘moderately energetic’ (e.g. dancing, cycling, leisurely swimming); and vigorous PA (e.g. running, hard swimming, squash), where active men and women had on average higher PF at follow-up, compared with their sedentary counterparts^(38)^. The results from the InCHIANTI study (men and women aged 65 years or older, *n* 1149) showed that physical inactivity was associated with decline in modified SPPB score and disability when compared with physically active participants^(36)^. In their study, participants were allocated to different groups for PA level.

The response categories were (i) minimal, (ii) light: performed 2–4 h/week not accompanied by sweating, (iii) moderate: performed 1–2 h/week accompanied by sweating or light PA not accompanied by sweating for more than 4 h/week, (iv) moderate PA: performed ≥3 h/week accompanied by sweating and (v) physical exercise: performed regularly that required maximal strength and endurance several times per week.

There is some evidence to suggest PA in interaction with higher protein intake^(39)^ which may be an effective strategy to prevent PF decline in older people, especially because inadequate protein intake is common in older adults, even among the physically active^(40)^. The Framingham Offspring observational study among middle-aged adults showed that active subjects with higher intakes of animal or plant protein source foods (red meat, poultry, fish, dairy products, soya, nuts, seeds and legumes) had higher skeletal muscle mass and a 35 % lower risk of functional decline^(41)^. However, dietary protein intake was not reported in the study. Results of our study detected several positive associations in the interaction of PA and protein intake with PF measures. However, we did not find an association between PA and protein intake interaction with changes in PF variables. This might be mostly due to the rather short follow-up period, which may have limited the ability of the present study to capture changes in PF. In addition, our study population age had a mean of 65 years and PF decline might not have been happening at a rapid pace at this age^(42)^. Furthermore, the full adjustment resulted in a loss of significance in the association of PA with PF measures, but not in the cumulative association of PA and protein intake with PF measures, suggesting a strong association between these two factors. There are no studies in literature why ordinary PA (including different household duties, gardening, climbing chairs, etc.) increases the demand for protein intake. More studies are needed to figure out the effectiveness of different physical activities to demand for protein and physical performance.

### Physical activity and interaction of physical activity and protein intake with muscle mass

Previous studies have shown the benefit of PA and exercise to increase muscle mass in older adults^(7,43,44)^. While PA was not associated with muscle mass, our results remained inconclusive regarding association of interaction of PA and protein intake, where highest value for LM and RSMI was detected among women with lowest PA and protein intake at the 3-year follow-up, to the contrary at baseline, highest value for RSMI was belonged to women with highest PA and protein intake. This may be explained by the argument that the ageing muscle loss of motor neurones may result in an increase in size of remaining motor units along with higher type 1 fibres preservation which means a possible preservation of muscle mass with relatively fewer type 2 fibres, thus lower strength. Our finding may suggest that higher PA and protein intake can be beneficial to PF measures in older adults regardless of preserving or increasing muscle mass^(33)^. However, this aspect is difficult to study without muscle biopsy, and further studies are warranted. Furthermore, our PA included household duties and other lighter PA which may not increase RSMI but could maintain or increase PF.

Subsequently, this finding may suggest that higher PA may improve PF and muscle strength when compared with muscle mass and it may show its benefit by reducing FM in older women. In a resistance training programme^(45)^, 10-m walking speed improvement after 8 week was associated with increased lower limb muscular strength and muscle quality, but not with muscle mass or body fat changes in older women^(45)^. Furthermore, studies in older people indicated that the decline in muscle strength exceeds the decline in mass^(46–48)^, and higher FM can predict lower muscle quality^(47)^. In this, data were also previously presented that a greater FM was adversely associated with multiple PF^(24)^. However, further studies are warranted to reveal the association between PA and protein intake with muscle quality rather than sheer body composition.

The effect of protein supplementation and exercise intervention has also been evaluated in clinical intervention studies. A recent meta-analysis of RCT regarding the effects of protein supplementation on the body composition and PF of older people undergoing resistance exercise training concluded that compared with resistance exercise training alone, protein supplementation combined with resistance exercise training may have a stronger effect in preventing ageing-related muscle mass attenuation and leg strength loss in older people^(49)^. However, the Society for Clinical and Economic Aspects of Osteoporosis, Osteoarthritis and Musculoskeletal Diseases working group noted in 2018 that protein supplementation intake has the potential to slow muscle mass loss, but evidence of the functional benefits of supplementation is mixed^(50)^. It appears that the interaction with protein supplementation intake and physical exercise requires further investigation. It is noteworthy that randomised control trials using protein supplementation and exercise intervention are mostly limited to a short follow-up period and may not be able to capture the effect of habitual PA and protein intake on PF and muscle mass in older adults. In addition, while multiple studies have been based on protein intake supplementation, a limited number of studies have used the habitual dietary intake of protein.

A novelty of the present study is that it provides the opportunity to investigate the association of PA independently and in combination with protein intake, according to WHO and NNR recommendations, respectively. We endeavoured to state previous studies which focused on the association of PA alone or by interaction with protein intake; however, it is important to note that PA is a term which has been used interchangeably with exercise or vice versa. We have mentioned the PA ascertainment method of each of these studies to provide easier comparison.

We used dual-energy X-ray absorptiometry measurement, which is a common tool suitable for the estimation of body composition in terms of evaluating the ratios of fat, muscle and bone in different parts of the body. Dual-energy X-ray absorptiometry has been shown to provide accurate estimations of body composition^(51)^. The percentage of frail people in these data was not considerably high. Among the subjects of the present study, according to the frailty phenotype definition by Fried *et al*., 8·1 % (*n* 36) of the women was classified as frail^(52)^.

PA data were collected via questionnaires, which has some limitation. For practical reasons, PA questionnaires are currently the most commonly used assessment method in large population-based cohort studies to assess individual PA levels. However, the agreement between different PA questionnaires in correctly revealing individuals as physically active (e.g. meeting the adult PA recommendations of >2·5 h/week) is challenging^(39)^. Another limitation of this method is over-reporting of PA, which may have led to an overestimation of subjects identifying themselves as sufficiently physical active when PA is assessed using self-reporting. In addition, capturing the intensity of PA by means of a questionnaire is susceptible to individuals’ perceptions, which can vary for a single individual or from person to person. Although all the questionnaires were checked by trained nurses, it may be that answers to the questionnaire regarding the intensity of PA refer more to health status and fitness level rather than strenuous PA. Therefore, the intensity of PA, as asked by the questionnaire in the present study, rather than exposure, was introduced as a covariate in the analysis models. It is recommended that further studies use PA validation methods to minimise such bias.

There are some other limitations to the present study that are important to take into consideration. For example, although the 3-d food record method has been described as a suitable instrument for assessing energy and protein intake in older people^(53)^, a repeated measure of 3-d dietary records at the follow-up could provide a more quantifiable measure to capture long-term protein intake. It is noteworthy that energy intake among our study population was relatively low, which may be due to underreporting (conscious or unconscious) or to actually reducing the typical level of food intake^(53)^. However, none of the participants met the threshold to be excluded from the analyses based on the BMR cut-offs indicated by the dietary reference values for food energy and nutrients for the UK^(54)^. Finally, we have controlled for several confounders, but the possibility of other confounding factors may exist.

It is worth noting that although RCT are of high value, they are not able to capture lifestyle-related variables such as diet, protein intake and PA. They are usually short by nature and are conducted in a controlled situation. Thus, observational studies such as this may provide information beyond RCT to reflect how habitual diet and PA may interactively be associated interactively with PF. Furthermore, a novel approach in the present study was to introduce the independent and interaction association of PA according to the WHO with protein intake according to NNR recommendations, which has previously not been studied.

### Conclusion

In conclusion, our findings suggest that higher PA, as recommended by the WHO, in combination with adequate protein intake, as recommended by NNR 2012, may have a positive association with better PF in older adults. However, further studies with a longer follow-up period are warranted to confirm this finding.

## Supplementary material

For supplementary material accompanying this paper visit http://dx.doi.org/10.1017/S0007114520000045. click here to view supplementary material
