# Supplementary material for: Interaction of recommended levels of physical activity and protein intake is associated with greater physical function and lower fat mass in older women: Kuopio Osteoporosis Risk Factor- (OSTPRE) and Fracture-Prevention Study
Source: Br J Nutr. 2020 Jan 8;123(7):826–39. doi: 10.1017/S0007114520000045 (PMC7054249; doi:10.1017/S0007114520000045)
Supplement: Supplementary file 1 [file S0007114520000045sup001.docx]

| Supplementary table 1. Correlation coefficients (r) between physical activity, protein intake and physical function in older women at the baseline. | | | | | | | | | | | |
| --- | --- | --- | --- | --- | --- | --- | --- | --- | --- | --- | --- |
|  | Grip strength | Grip strength/BMI | Knee extension | Standing up from chair | Tandem walk speed 6m | Walking speed 10m | Standing on one leg | Ability to perform squat | Modified SPPB | Lean mass | RSMI |
| Physical activity  n=608 | -0·004 (0·931) | 0·041 (0·325) | 0·116 (0·007) | -0·013 (0·754) | 0·089 (0·050) | 0·171 (0·001) | 0·146 (0·001) | 0·121 (0·004) | 0·121 (0·004) | -0·020 (0·618) | -0·021 (0·599) |
| Protein intake  n=554 | -0·090 (0·134) | 0·138 (0·001) | 0·006 (0·894) | 0·035 (0·407) | 0·052 (0·225) | 0·211 (0·001) | 0·125 (0·001) | 0·157 (0·001) | 0·172 (0·001) | -0·229 (0·001) | -0·239 (0·001) |
| Physical activity * protein intake  n=554 | 0·009 (0·565) | 0·181 (0·001) | 0·117 (0·006) | 0·056 (0·184) | 0·112 (0·006) | 0·217 (0·001) | 0· 237 (0·001) | 0· 239 (0·001) | 0·217 (0·001) | 0·069 (0·576) | -0·053 (0·208) |
| Values are *Pearson’s correlation coefficients (p-values).* | | | | | | | | | | | |
